# Insights into the geometries, electronic and magnetic properties of neutral and charged palladium clusters

**DOI:** 10.1038/srep19656

**Published:** 2016-01-22

**Authors:** Xiaodong Xing, Andreas Hermann, Xiaoyu Kuang, Meng Ju, Cheng Lu, Yuanyuan Jin, Xinxin Xia, George Maroulis

**Affiliations:** 1 Institute of Atomic and Molecular Physics, Sichuan University, Chengdu 610065, China; 2 Department of Physics, Nanyang Normal University, Nanyang 473061, China; 3 Centre for Science at Extreme Conditions and SUPA, School of Physics and Astronomy, The University of Edinburgh, Edinburgh EH9 3JZ, United Kingdom; 4 Beijing Computational Science Research Center, Beijing 100084, China; 5 Department of Chemistry, University of Patras, GR-26500 Patras, Greece

## Abstract

We performed an unbiased structure search for low-lying energetic minima of neutral and charged palladium Pd_*n*_^*Q*^ (*n* = 2–20, *Q* = 0, + 1 and –1) clusters using CALYPSO method in combination with density functional theory (DFT) calculations. The main candidates for the lowest energy neutral, cationic and anionic clusters are identified, and several new candidate structures for the cationic and anionic ground states are obtained. It is found that the ground state structures of small palladium clusters are more sensitive to the charge states. For the medium size Pd_*n*_^0/+/–^ (*n* = 16–20) clusters, a fcc-like growth behavior is found. The structural transition from bilayer-like structures to cage-like structures is likely to occur at *n* = 14 for the neutral and cationic clusters. In contrast, for the anionic counterparts, the structural transition occurs at Pd_13_^–^. The photoelectron spectra (PES) of palladium clusters are simulated based on the time-dependent density functional theory (TD-DFT) method and compared with the experimental data. The good agreement between the experimental PES and simulated spectra provides us unequivocal structural information to fully solve the global minimum structures, allowing for new molecular insights into the chemical interactions in the Pd cages.

There is active interest in the synthesis and electrochemical applications of palladium-based nanomaterials^1^. Of particular commercial potential is the replacement of platinum-based electrocatalysts with palladium-based ones. Overall, palladium clusters have attracted considerable attention in recent years. Fields of particular interest are hydrogen storage^2^, catalysis^3^,^4^, sensors^5^ and biomedicine^6^. Clusters are considered a new and challenging kind of molecular architecture, enabling the transition from few-atom assemblies, to nanoparticles and the bulk. Molecular science is facing unique problems, especially in the determination of stable structures and therefore explanations of the variation of the physicochemical properties. Several experiments^7^,^8^,^9^ confirm that small palladium clusters exhibit a novel ferromagnetism, although there is no magnetic moment both in the atomic state and in the bulk, due to the closed shell electronic (4*d*^10^5*s*^0^) configuration of the atom. Recent breakthrough work^10^,^11^,^12^ clearly suggests that synthesized subnanometer palladium clusters can be stabilized. In practice, they exhibit enhanced catalytic properties in synthesis and *in situ* characterization methods of ultra-small metal clusters. Understandably, the stability of the molecular architectures and their electronic configuration is seen as a very important in the study of the ground state of palladium clusters.

The growth behavior and respective patterns of palladium clusters have been systematically studied. Kumar *et al*.^13^ presented an icosahedral growth pattern for neutral palladium clusters (*n* = 2–23, 55 and 147). Futschek *et al*.^14^ investigated the structure of small Pd_*n*_ (*n* = 2–13) clusters and suggested that the favorable structures of Pd_11_, Pd_12_ and Pd_13_ clusters are the fcc fragments of bulk palladium. Zhang *et al*.^15^ examined closely four types of possible structural motifs (fcc like, decahedron based, icosahedron based and prolate ellipsoid-based) and advanced the view that medium-sized Pd_*n*_ (*n* = 15–25) clusters prefer fcc-like structures. The case of the Pd_13_ cluster merits particular attention. Reddy *et al*.^16^ suggested the ground state of this cluster favors a compact packing. A lower energy buckled biplanar structure was predicted by Chang and Chu^17^. An even lower energy structure than the above was found in a distorted hexagonal bilayer-like structure by Piotrowski *et al*.^18^. Subsequently, through an integrated theoretical and experimental analysis, a bilayer ground-state structure was determined by Köster *et al*.^19^ Two more studies are particularly interesting. Nava *et al*.^20^ presented a reference study on neutral, large Pd_*n*_ (*n* = 2–309) clusters; and the average bond length in Pd_*n*_ (*n* = 4–309) clusters was investigated by Krüger *et al*.^21^

Most of the theoretical studies focus on neutral palladium clusters. Few studies are available for the cationic and anionic species^22^,^23^,^24^. We deem essential a systematic investigation of neutral and charged medium-sized palladium clusters Pd_*n*_^0/+/–^ (*n* = 2–20). The present study on medium-sized clusters is not restricted to molecular geometries and stabilities but also investigates electric and magnetic properties. In addition, we have calculated for the first time the relevant photoelectron spectra. As these are directly comparable to experimental data, this comparison constitutes a valuable test of the reliability of our computational strategy.

The central aim of our work is the systematic, in-depth study of the structural evolution and properties of palladium (neutral, cationic and anionic) clusters by combining the unbiased CALYPSO method with density functional theory techniques. Thus, we are able to advance a fundamental understanding of the ground state geometric configurations of neutral, cationic and anionic Pd_*n*_^0/+/–^ (*n* = 2–20) clusters. Subsequently, we reexamine carefully the structure of specific neutral, cationic and anionic low-energy isomers of palladium clusters that have been the target of previous investigations by other workers. Finally, we explore closely the physical mechanism of the growth behavior of palladium clusters and provide new insights that should be of importance to further theoretical and experimental studies. The paper is organized as follows: we present and discuss the findings pertaining to geometrical structures, photoelectron spectra, relative stabilities, magnetic properties and polarizabilities of the ground state structures in the following. Then, our conclusions are summarized. Finally, details of the computational method are concisely described.

## Results and Discussion

### Geometric structures

**Figure 1:**
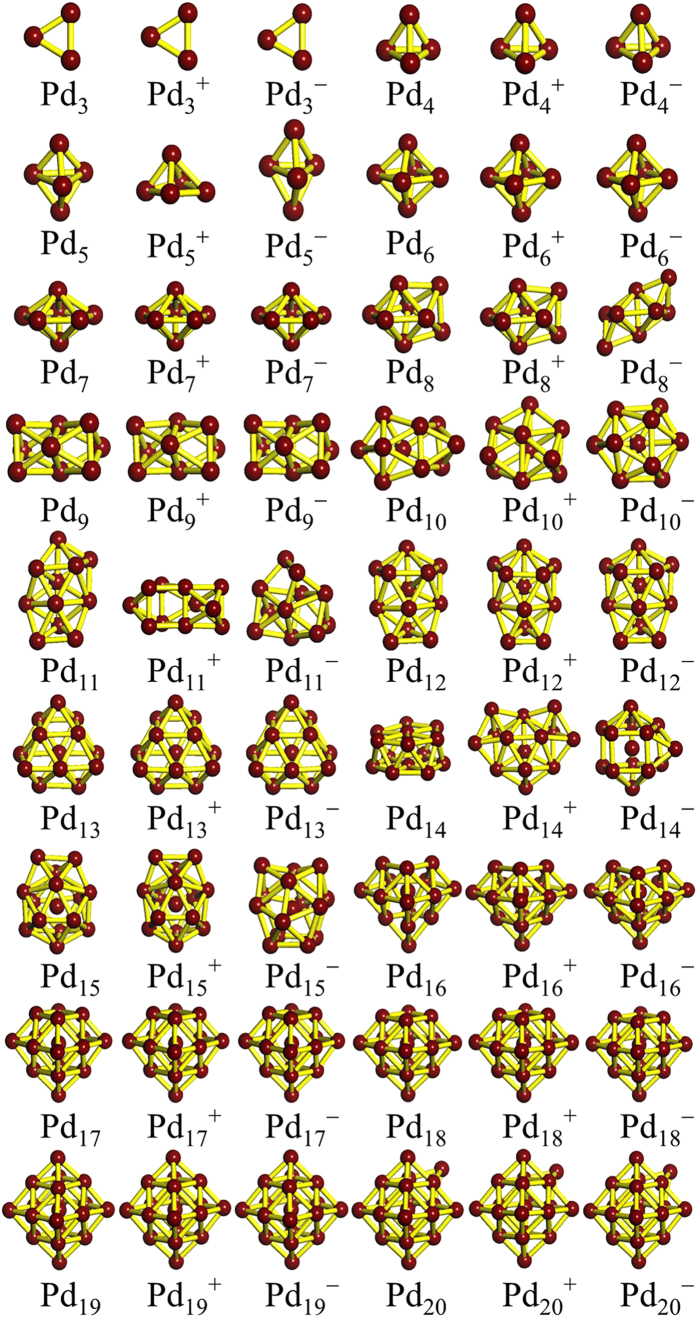
Lowest-energy structures of Pd_*n*_^0/+/–^ (*n* = 3–20) clusters.

The global minimum structures for the lowest energy neutral and charged palladium clusters in the size range of *n* = 2–20 are displayed in Fig. 1. Previously reported isomers, from experiment and theory, are successfully reproduced with our structure search method. Representative metastable isomers of neutral and charged clusters, together with their point group symmetries and relative energies are displayed in Figs S1–S3 in the Supporting Information (SI). The corresponding electronic state, point group symmetry, binding energy (*E*_*b*_) and HOMO−LUMO gap (*E*_*gap*_) of the ground state structures are also determined and listed in Table S1 (see SI).

Previous findings^22^,^24^ on small (*n* ≤ 7) clusters revealed that the neutral and corresponding charged palladium clusters have similar geometric configurations and follow the same structural evolution. The respective ground state structures found in our searches are in accord with those previously reported experimental and theoretical findings, which corroborates the reliability of our theoretical method. We then carefully investigate small Pd_*n*_^*Q*^ clusters to pinpoint the characteristic transformations brought by the addition or removal of an electron. In the case of *n* = 2–4, 6 and 7, neutral palladium clusters often have the same or quite similar structures to the corresponding charged palladium clusters. A striking structural transformation occurs at *n* = 5. Neutral and anionic Pd_5_^0/–^ clusters have similar trigonal bipyramid structures, while the cationic Pd_5_^+^ cluster is a pyramid with a bent base. It is worth mentioning that the new structure of cationic Pd_5_^+^ is 0.06 eV lower in energy than the trigonal bipyramid structure reported by Kalita *et al*.^22^.

Although such small Pd_*n*_^*Q*^ clusters have been extensively studied, relatively few studies are available for medium or large size Pd_*n*_^*Q*^. As is well known, the true ground state structures for neutral palladium clusters in the range of *n* = 8–20 have been the object of some controversy. The marked disagreement between early studies confirms the existing difficulty in reliably identifying the structural characteristics of medium and large Pd_*n*_^*Q*^ clusters. Turning our attention to the range of *n* = 8–20, we observe a fcc-like growth pathway for neutral Pd_*n*_ (*n* = 16–20) clusters, in agreement with the related findings of Zhang *et al*.^15^ and Mu *et al*.^25^ For charged clusters, we note a significant structural transformation. This structural evolution of medium-sized charged Pd_*n*_^+/–^ (*n* = 16–20) clusters has, to our knowledge, not been noticed before. Neutral and charged clusters display three entirely different structures for *n* = 10, 11, and 14. For *n* = 10 and 11, the charged clusters favor corresponding distorted neutral structures. It is clear that the neutral Pd_14_ cluster favors a bilayer-like structure with two face centered hexagonal surfaces. This configuration lies 0.13 eV lower than the cage-like structure with three capped atoms of the metastable cluster in Fig. S1 (see SI). The cationic Pd_14_^+^ cluster is most stable in a face-centered bilayer-like configuration that has not been reported previously. In the case of the anionic Pd_14_^–^ cluster, the ground state structure is found to be the cage-like structure that is the metastable Pd_14_ structure. The anionic Pd_14_^–^ cluster is thus the first cage-like structure amongst Pd clusters as the size of the clusters increases. More generally, all neutral and charged clusters can be viewed as cage-like structures for *n* ≥ 15. It is safe to conclude that for medium-sized palladium clusters there exists a structural transition point from bilayer-like structures to cage-like structures. The transition occurs at *n* = 14 for the neutral and cationic clusters and at *n* = 13 for the anionic cluster.

As discussed above, we find that small palladium clusters are more sensitive to the charge state than the larger clusters. Several new lowest-energy structures are identified and shown here for cationic and anionic clusters. These promising candidates have never been reported as ground state structures. To the best of our knowledge, there are few findings about medium-sized cationic and anionic palladium clusters to compare with ours or to be considered for a reexamination. Hence, our results for these clusters should provide a valuable basis for further investigations.

### Photoelectron spectra

To verify the accuracy of the ground state structures of palladium clusters we have calculated the adiabatic detachment energies (ADEs = *E*_optimized neutral_ – *E*_optimized anion_) and the vertical detachment energies (VDEs = *E*_neutral at optimized anion geometry_ – *E*_optimized anion_). The computed results are summarized in Table 1, along with available theoretical and experimental values. As shown in Table 1, the calculated ADE and VDE values are in good agreement with the experimental values^26^,^27^ for all cases with the notable exception of *n* = 3. There, the discrepancy between our theoretical and experimental values is almost 0.50 eV, but our calculations agree well (deviate by only 0.14 eV) with other theoretical results^24^. For the larger size clusters (*n* = 16–20), unfortunately, no experimental data are available to compare with our results.

**Figure 2:**
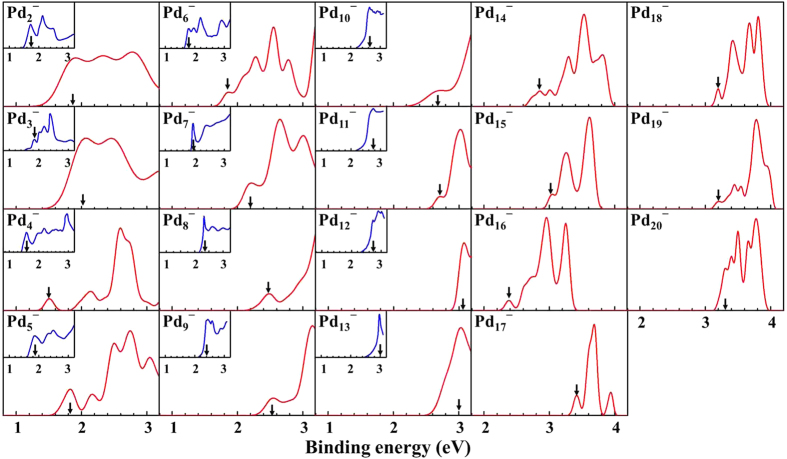
The calculated photoelectron spectra of Pd_*n*_^–^ (*n* = 2–20) clusters, together with the available experimental data for comparison. The first major peaks are marked with vertical arrows. The experimental PES of Pd_*n*_^–^ (*n* = 3–8) clusters come from ref. 26, while the rest of experimental PES come from ref. 27.

A deeper understanding of the cluster’ electronic properties is obtained through the simulation of the photoelectron spectra (PES) of anionic palladium clusters (*n* = 2–20) as obtained using TD-DFT. The theoretical along with the experimental PES spectra^26^,^27^ are shown in Fig. 2. In this work, the full width at half maximum of Gaussian function of anionic Pd_*n*_^–^ (*n* = 2–20) clusters is in the range of 0.05–0.40 eV. Restricted by experimental conditions, the overall experimental PES is mainly used to estimate the position of the first strong maximum peak. From an experimental viewpoint, the binding energy of first major peak represents the vertical detachment energy (VDE). In particular, the experimental PES for anionic Pd_*n*_^–^ (*n* = 7–13) clusters show only one prominent peak in the region 1.0–3.0 eV. To make the comparison with experimental PES, we align the VDE of simulated spectra with the experimental spectra. From Fig. 2, the VDE values of simulated PES are found to be in quite good agreement with the experimental data for anionic clusters (*n* = 2–13), while there is a discrepancy for *n* = 3. This may be due to that palladium has a variety of isotopes (^102^Pd, ^104^Pd, ^105^Pd, ^106^Pd, ^108^Pd and ^110^Pd), which increase the difficulty of precise measurement for experimental PES. The theoretical PES for the anionic clusters (*n* = 16–20) show several recognizable peaks in the range of binding energy 3.0–4.0 eV. In general, the PES data should also provide a definitive electronic fingerprint for the ground state structures. We hope that the theoretical PES values together with the calculated ADE and VDE of Pd_*n*_^–^ (*n* = 16–20) clusters will provide valuable information for future experimental investigations.

### Relative stability

The inherent stability of the palladium clusters studied in this work is determined by computing the binding energy per atom (*E*_*b*_), defined as


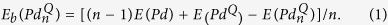


**Figure 3:**
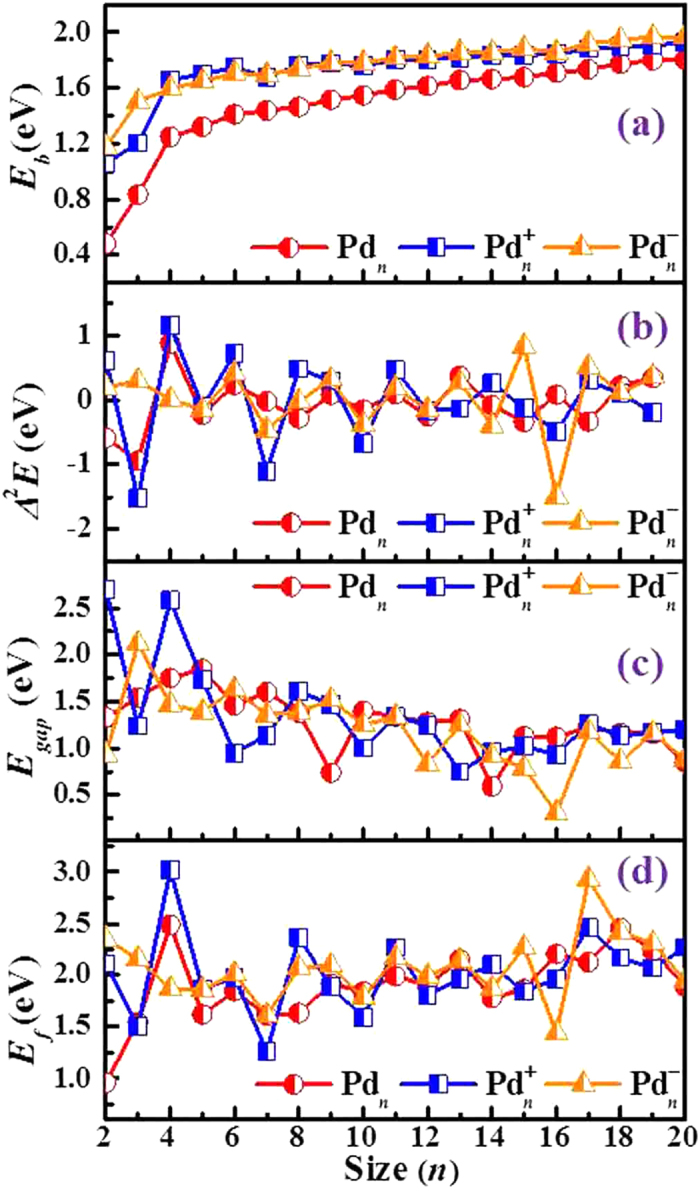
The binding energy per atom (*E*_*b*_), second energy difference (∆^2^*E*), HOMO–LUMO gap (*E*_*gap*_), and fragmentation energies (*E*_*f*_) of the preferred fragmentation channels for the lowest–energy Pd_*n*_^0/+/–^ (*n* = 2–20) clusters.

*E* is the total energy of the clusters. The average binding energy (*E*_*b*_) of a cluster is a measure of its thermodynamic stability. For Pd_*n*_^0/+/–^ clusters, the calculated results of *E*_*b*_ as a function of cluster size *n* are displayed in Fig. 3(a). As evidenced from Fig. 3(a), the *E*_*b*_ values of neutral Pd_*n*_ cluster are always much lower than their charged counterparts. In addition, the *E*_*b*_ values of neutral and charged clusters increase monotonically with cluster size, except for a dip found for cationic and anionic clusters at *n* = 7. As expected, the difference between the binding energy of clusters and bulk gets smaller with increasing cluster size, and the average binding energy approaches the cohesive energy of 3.89 eV/atom of bulk palladium^28^.

Another important parameter to measure the relative stabilities of the most stable clusters is the size-dependent second energy difference (∆^2^*E*), which can be calculated by


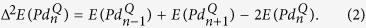


The calculated results of ∆^2^*E* as a function of cluster size are shown in Fig. 3(b). It is well-known that a maximum of ∆^2^*E* relative to its immediate neighbors indicates an enhanced stability of a particular cluster. Therefore, the values of ∆^2^*E* can be considered as an alternative measure of the stability of clusters, where the most stable clusters are identified from the maxima of ∆^2^*E*. Figure 3(b) reveals conspicuous odd-even oscillations for each state. For neutral clusters, the odd size clusters show a greater stability compared to their even counterparts for Pd_*n*_ (7 < *n* < 15) clusters. Moreover, several pronounced peaks are found at *n* = 4, 6, 9, 11, 13 and 16, implying strong relative stability. A similar oscillatory trend is found for anionic Pd_*n*_^–^ clusters in the size range of *n* > 8, while cationic Pd_*n*_^+^ (2 ≤ *n* ≤ 8) clusters present an opposite oscillating behavior. In addition, some local maxima of ∆^2^*E* are observed at *n* = 4, 6, 8, 11, 14 and 17 for cationic clusters as well as *n* = 3, 6, 9, 11, 13, 15 and 17 for anionic clusters.

The electronic energy gap (*E*_*gap*_) reflects the energy cost for an electron to jump from the highest occupied molecular orbital (HOMO) to the lowest unoccupied molecular orbital (LUMO). A large *E*_*gap*_ usually correlates with remarkable kinetic stability. The calculated values of HOMO–LUMO energy gaps for the ground state of Pd_*n*_^0/+/–^ (*n* = 2–20) clusters are listed in Table S1. We plot the *E*_*gap*_ values as a function of cluster size *n* in Fig. 3(c). It is interesting to notice that the curves for neutral and charged clusters show a fluctuant downward trend, indicating that small clusters are more stable than the larger clusters. At the same time, we find some conspicuous peaks, Pd_5_, Pd_7_, Pd_10_, Pd_13_, Pd_17_ and Pd_19_ for neutral clusters, Pd_4_^+^, Pd_8_^+^, Pd_11_^+^, Pd_15_^+^ and Pd_17_^+^ for cationic clusters, as well as Pd_3_^–^, Pd_6_^–^, Pd_9_^–^, Pd_11_^–^, Pd_13_^–^, Pd_17_^–^ and Pd_19_^–^ for anionic clusters. These peaks reflect stronger local stability of the clusters compared to their neighbors.

**Figure 4:**
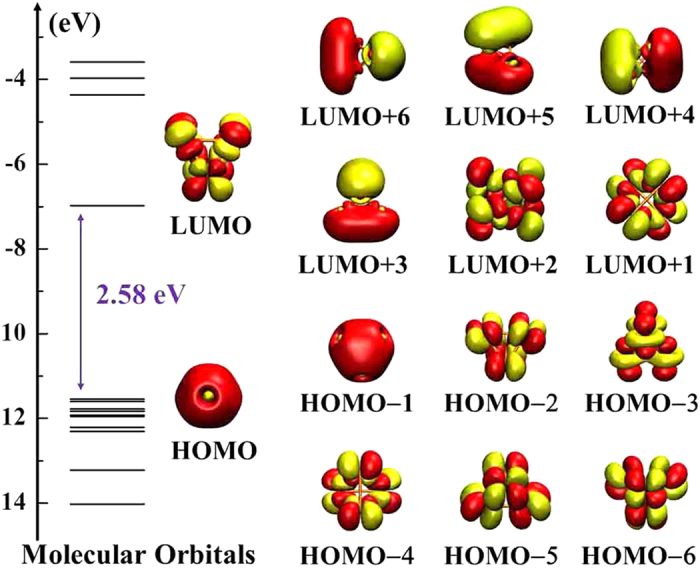
One-electron energy levels and molecular orbital wave function isosurfaces (isovalue = 0.02 au) for Pd_4_^+^.

Note that the Pd_4_^+^ cluster corresponds to a maximum of the ∆^2^*E*, the second energy difference, but also to a remarkable peak of *E*_*gap*_, the HOMO–LUMO gap. This suggests its stability among neutral and charged clusters. We have calculated the one-electron energy levels and the corresponding molecular orbitals for the Pd_4_^+^ cluster (see Fig. 4). Despite their different shapes, there are three kinds of orbitals: *s*-type molecular orbital, *p*-type molecular orbital and *d*-type atomic orbital. We also observe that *d*-type atomic orbital dominate the molecular orbitals diagram of the cluster. From Fig. 4, we infer that the HOMO and LUMO of Pd_4_^+^ occupy an *s*-type molecular orbital and *d*-type atomic orbital, respectively. Regarding the lower occupied levels, the HOMO-1 possesses *s*-type molecular orbital, while HOMO-2 to -6 resemble *d*-type atomic orbitals. The LUMO + 1 and LUMO + 2 levels resemble *d*-type atomic orbitals, while higher LUMO + *q* (*q* = 3–6) levels occupy *p*-type molecular orbitals. In addition to *T*_*d*_ symmetry, the Pd_4_^ + ^cluster displays characteristic degeneration which may give rise to the largest *E*_*gap*_ (2.58 eV).

**Figure 5:**
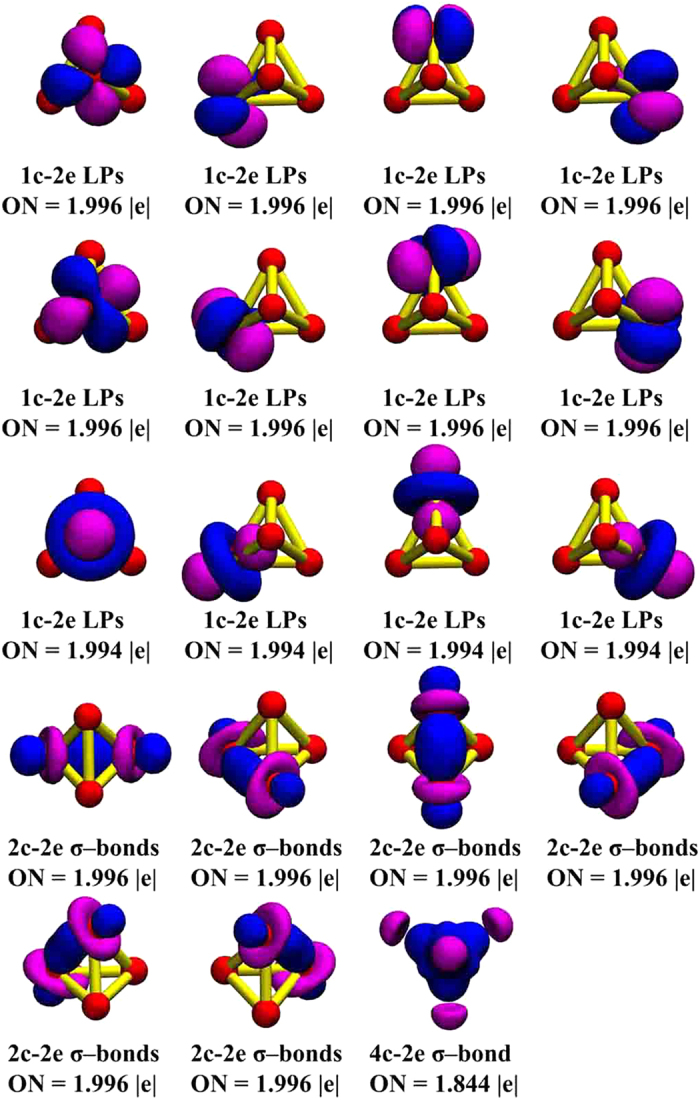
Chemical bonding analysis of Pd_4_^+^ cluster using the AdNDP method. ON stands for occupation number.

We performed a chemical bonding analysis based on the localized orbitals resulting from the AdNDP algorithm. The AdNDP approach is an ideal tool for deciphering the nature of bond localization and delocalization in clusters; in short it takes advantage of the electron pair as the unit of chemical bonding by partitioning the electron density matrix into *n*c−2e terms (*n* ranges from 1 to the maximum number of atomic centers). The AdNDP analysis of Pd_4_^+^ cluster is shown in Fig. 5. The AdNDP analysis indicates eight lone pairs (LPs) with an occupation number ON = 1.996 |e|, four other LPs with ON = 1.994 |e|, six 2c–2e Pd-Pd σ bonds with ON = 1.996 |e| and one 4c–2e σ bond with lower ON = 1.844 |e|. It is interesting to note that each atom has three lone pair localized bonds.

To further advance our understanding of the dynamical stability of the Pd_4_^+^ cluster, show its vibrational IR and Raman spectra in Fig. S4 (see SI). The insets show the eigenvectors of the Pd_4_^+^ cluster for the modes with the highest IR intensity or Raman activity. It is worth noting that both can be considered as spectroscopic fingerprints to confirm the existence of Pd_4_^+^ cluster between different species and different isomers in future experiments.

### Fragmentation channels

It is known that fragmentation processes may involve dissociation barriers. However, from a purely energetic point of view, the fragmentation energies (*E*_*f*_) of the most stable clusters can be expressed as


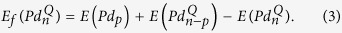


In this work, we have considered all possible fragmentation channels for neutral and charged clusters and the respective values for *E*_*f*_ are listed in Table S2–S4 (see SI). By definition, the preferred dissociation channels are those yielding the lowest *E*_*f*_^29^. In the case of negative *E*_*f*_ for particular fragmentation channels, the initial clusters are unstable and could dissociate spontaneously by releasing a **|***E*_*f*_**|** amount of energy. In our calculations, the energy of all possible fragmentation channels is positive, indicating that the initial clusters are stable and must absorb energy to induce the fragmentations. The Pd_*n*_^0/+/–^ → Pd_*n*-1_^0/+/–^ + Pd channels are then the routes favored by all palladium clusters (*n* = 2–20). I.e., the neutral and charged palladium clusters tend to lose individual atoms when absorbing energy.

The *E*_*f*_ values for the respective most favorable fragmentation channels are plotted as a function of cluster size in Fig. 3(d). Highest stability, associated with an energy maximum, is evidenced for Pd_4_ for neutrals and Pd_17_^–^ for anions as well as Pd_4_^+^ for cations. Moreover, several local maximums compared to their neighbors are observed: Pd_6_, Pd_9_, Pd_11_, Pd_13_, Pd_16_ and Pd_18_ for neutrals, Pd_6_^+^, Pd_8_^+^, Pd_11_^+^, Pd_14_^+^ and Pd_17_^+^ for cations, and Pd_6_^–^, Pd_11_^–^, Pd_13_^–^ and Pd_15_^–^ for anions. It should be stressed again that the Pd_4_^+^ cluster is characterized by remarkable stability. This is in accordance with the above analysis based on the second energy difference ∆^2^*E* and the energy gap *E*_*gap*_. As a consequence, an analysis of the ∆^2^*E*, *E*_*gap*_ and *E*_*f*_ values, provides the following magic numbers: (i) *n* = 13 for neutral clusters; (ii) *n* = 4, 8, 11 and 17 for cationic clusters; (iii) *n* = 6, 11, 13 and 17 for anionic clusters.

### Magnetic properties

**Figure 6:**
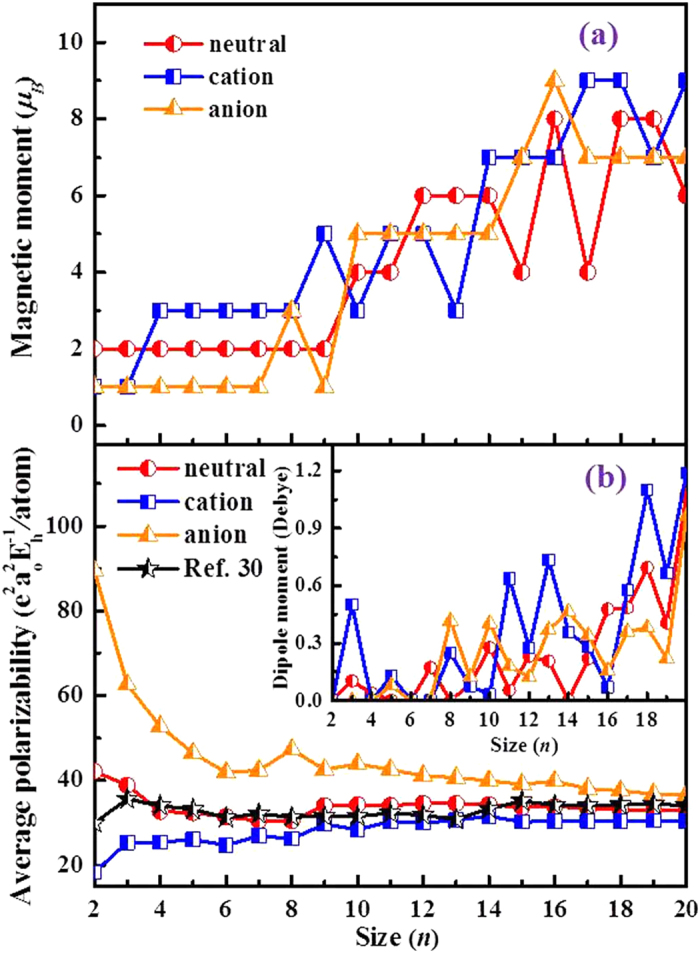
(**a**) Size dependence of spin magnetic moments (*μ*_*B*_) for the lowest-energy structures of Pd_*n*_^0/+/–^ (*n* = 2–20) clusters. (**b**) The electric dipole moment (*μ*) and mean polarizability per atom (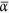/*n*) of Pd_*n*_^0/+/–^ (*n* = 2–20) clusters, together with the available theoretical data for comparison.

The total spin magnetic moments of Pd_*n*_^0/+/–^ clusters as a function of cluster size *n* are shown in Fig. 6(a). In addition to several peaks and troughs, a typical step-like behavior of the total magnetic moment is observed in the lowest-energy state of the neutral clusters, a fact previously reported by other research groups^13^,^25^,^28^. A very similar situation is also observed for cationic and anionic clusters. We regard this striking variation as a consequence of the difference of their geometrical configurations, as the magnetic energy is extremely sensitive to minor geometry changes^28^. For the sake of comparison, the magnetic moment per atom (*μ*_*B*_ /atom) is calculated and compared to theoretical values by Kumar *et al*.^13^ in Table 1. It is worth mentioning that there is a consensus in the recent theoretical literature^15^,^25^ that the highly symmetric icosahedral structures tend to possess large magnetic moments as reported by Kumar *et al*.^13^ Although the results of neutral palladium clusters agree very well with those of Kumar *et al*.^13^ at *n* < 8, the various structures of Pd_*n*_ (*n* = 8–15) clusters clearly favor lower magnetic moment values. Cox *et al*.^7^ reported that Pd_13_ has a magnetic moment of ~ 0.4 *μ*_*B*_ at 98 K. This is in excellent agreement with our theoretical value of 0.46 *μ*_*B*_ at absolute zero. From a purely theoretical point of view, below the Curie temperature an increase in temperature leads to a decrease of the magnetic moment. Our computed value is reasonable. As we mentioned earlier, the bulk magnetic moment of palladium is zero, leading us to expect a decrease of the magnetic moment with increasing cluster size.

**Figure 7:**
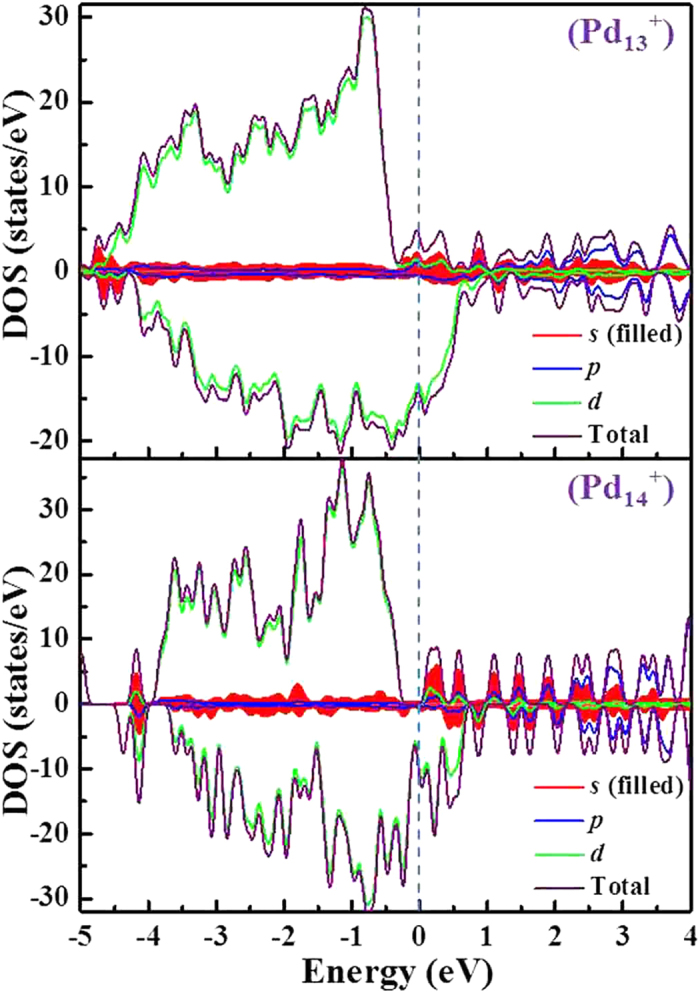
The density of states (DOS) of Pd_13_^+^ and Pd_14_^+^ clusters. The Fermi level is shifted to zero.

In order to probe the origin of the magnetic step-like behavior of palladium clusters, we analyze the total density of states (TDOS) and the projected density of electronic states (PDOS). Because a large magnetic moment jump occurs at *n* = 14 for cationic clusters, the Pd_13_^+^ and Pd_14_^+^ clusters are examined and shown in Fig. 7. As evidenced by the diagram, extremely strong hybridization occurs between *s*, *p* and *d* states. By comparing the results of TDOS and PDOS of Pd_13_^+^ and Pd_14_^+^ clusters, it is observed that the total magnetic moments mainly come from *d* states below the Fermi level, while the *s* and *p* states produce nearly negligible contributions. However, an obvious hybridization between *s*, *p* and *d* states is observed for Pd_13_^+^ and Pd_14_^+^ clusters in the vicinity of the Fermi level. We also notice that the up-spin and down-spin sub-bands of the *d* states are substantially split when going from *n* = 13 to *n* = 14. This may result in an enhanced magnetic moment. This conclusion can is corroborated by the TDOS and PDOS of anionic Pd_9_^–^ and Pd_10_^–^ as well as neutral Pd_15_ and Pd_16_ clusters, as plotted in Fig. S4 (see SI).

### Polarizabilities

The electric dipole moment and polarizability are closely related to the structural features and electronic properties of clusters. In terms of applications, the site-specific polarizabilities can be used to predict the optimal adsorption sites of an adsorbed molecule^29^. In this paper, we have calculated the mean polarizability per atom which is defined in terms of the components as 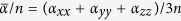, where *n* is the number of atoms in the cluster. The calculated results and the electric dipole moments (*μ*) of the neutral and charged ground states are depicted in Fig. 6(b). The mean polarizability per atom and electric dipole moments of the neutral and charged clusters are also listed in Table S5 and S6 (see SI). On account of their high symmetries, the dipole moments of several clusters (Pd_2_^0/+/–^, Pd_4_^+/–^, Pd_6_^+/–^ and Pd_7_^+/–^) vanish. Notably, the addition or removal of an electron leads, as is the case of *n* = 4, 6 and 7, to significantly higher symmetry. This is the reason why the dipole moment of the charged clusters is zero. Furthermore, we note that our calculated polarizabilities for neutral and charged clusters are independent (see Fig. 6(b)). For the anionic clusters, the calculated 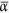/*n* decrease monotonously with the cluster size *n*, with the notable exception of a peak occurring at *n* = 8. The presence of this peak is attributed to the lower symmetry of *n* = 8 compared to the high symmetry of Pd_*n*_^–^ clusters in the range of *n* = 2–7. For the neutral clusters, owing to the different spin multiplicity, there is an apparent conflict compared to the singlet state value of Pd_2_ reported by Ma *et al*.^30^. As it is well established, relevant experiment findings^31^ and theoretical results^22^,^32^ point to a triplet for the ground state of diatomic palladium. So our result is more accurate. It is worth noting that earlier work by Li *et al*.^33^ reported mean dipole polarizability values of 58.3 (singlet) and 83.7 (triplet) e^2^a_0_^2^E_h_^–1^ for Pd_2_ (atomic units, 1 e^2^a_0_^2^E_h_^–1^ = 1.648778 × 10^–41^ C^2^m^2^J^–1^). Relying on a recommended theoretical value^34^ for the polarizability of the atom, *α*(Pd) = 32 e^2^a_0_^2^E_h_^–1^, we find that the differential-per-atom mean polarizability (DPA)^35^,^36^, defined for the Pd_*n*_ cluster as


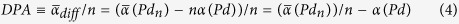


reaches a high value for the dimer: DPA(Pd_2_) ≈ 10 e^2^a_0_^2^E_h_^–1^. In the case of cationic clusters, we observe an upward fluctuant trend from *n* = 2 to *n* = 14 as well as an evident jump at *n* = 3. The value of 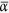/*n* for cationic clusters is around 30.4 e^2^a_0_^2^E_h_^–1^ in the range of *n* = 15 to 20. In addition, we observe a clear convergence of the mean polarizability per atom 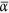/*n* of neutral and charged clusters for relative large cluster sizes.

## Conclusions

On the basis of the unbiased CALYPSO structure searching method and density functional theory calculations, we report a detailed investigation of neutral and charged palladium clusters. The most stable structures of the neutral clusters are compared with those of the charged clusters in the size range up to 20 atoms. The structure searches show that the ground state structures of small palladium clusters are more sensitive to the charge states than previously thought. A fcc-like growth behavior is found for the medium size Pd_*n*_^0/+/–^ (*n* = 16–20) clusters. The photoelectron spectra of Pd_*n*_^–^ clusters are simulated based on TD-DFT and compared with experimental data. The reasonable agreement between them indicates that our structure searches have very likely uncovered the actual ground state structures. The electronic and magnetic properties as well as fragmentation channels for the ground state Pd_*n*_ (*n* = 2–20) clusters are also studied in detail for both neutral and charged Pd clusters. The lowest-energy tetrahedral structure of Pd_4_^+^ cluster with *T*_*d*_ symmetry shows very high stability. The Pd_*n*_^0/+/–^ → Pd_*n*_^0/+/–^ + Pd channels are the favored fragmentation routes for all neutral, cationic and anionic clusters. Interestingly, a step-like magnetic behavior is observed for neutral and charged palladium clusters.

## Methods

We performed an unbiased structure search for the lowest energy neutral, cationic and anionic palladium clusters in the size range of *n* = 2–20, respectively. We employed the CALYPSO (Crystal structure AnaLYsis by Particle Swarm Optimization) searching method^37^,^38^ coupled with DFT calculations for geometry optimization. This combined CALYPSO/DFT computational approach has been previously used to search for low-lying sulphur clusters^39^, boron clusters^40^, and boron doped silver clusters^41^. Within the CALYPSO structure search, we adopted the global version of the particle swarm optimization (PSO) algorithm to finely explore the potential energy surface for each cluster size. This method has been successfully used to predict correctly ground state structures for various systems^42^,^43^,^44^,^45^. In the CALYPSO structure search, the initial structures are randomly generated with the constraint of symmetries^42^. When a new structure is generated, the bond characterization matrix is calculated to check the similarity of this structure with others. After all the structures have been generated, the local structural optimizations are performed to eliminate the noise of energy surface and drive the systems to the local minima. We typically followed 30 generations to generate at least 1000 structurally different low-energy isomers for each size. Among the 1000–1200 isomers, the top fifty low-lying isomers are collected as candidates for the lowest-energy structure. Those structures with energy difference from the lowest-lying isomers less than 0.3 eV (usually about 20) are further optimized to identify the lowest-energy structure. The geometry optimizations are performed without symmetry constraints using the Gaussian 09 package^46^ and the B3LYP (Becke three-parameter-Lee–Yang–Parr)^47^,^48^ hybrid exchange-correlation functional. The pseudopotential relativistic LANL2DZ^49^ basis set for Pd is employed in all calculations. Harmonic vibrational frequencies are calculated at the same level of theory in order to ensure that the obtained optimized structures are true minima (no imaginary frequencies). The photoelectron spectrum of the anionic palladium clusters is simulated by calculating excited energies within the framework of time-dependent density functional theory (TD-DFT)^50^. The chemical bonding analysis presented here is obtained via the adaptive natural density partitioning (AdNDP) method^51^,^52^.

## Additional Information

**How to cite this article**: Xing, X. *et al*. Insights into the geometries, electronic and magnetic properties of neutral and charged palladium clusters. *Sci. Rep*. **6**, 19656; doi: 10.1038/srep19656 (2016).

## Supplementary Material

Supplementary Information

## Figures and Tables

**Table 1:** The calculated adiabatic detachment energy (ADE, eV), vertical detachment energy (VDE, eV) and average magnetic moment per atom (*μ*_*B*_/atom) for palladium clusters, together with the available experimental and theoretical data for comparison.

Size	ADE		VDE			Average magnetic moment per atom			
						Neutral		Cation	Anion
	This work	Exp.	This work	Theo.	Exp.	This work	Theo.		
2	1.41	1.30 ± 0.15^a^	1.75	1.59^b^	1.71^a^	1.00	1.00^d^	0.50	0.50
3	2.02	1.35 ± 0.10^a^	2.04	1.90^b^	1.54^c^	0.67	0.67^d^	0.33	0.33
4	1.40	1.35 ± 0.10^a^	1.49	1.48^b^	1.59^c^	0.50	0.50^d^	0.75	0.25
5	1.64	1.45 ± 0.10^a^	1.94	1.57^b^	1.74^c^	0.40	0.40^d^	0.60	0.20
6	1.80	1.65 ± 0.10^a^	1.89	1.69^b^	1.82^c^	0.33	0.33^d^	0.50	0.17
7	1.80	1.70 ± 0.15^a^	2.13	1.81^a^	1.98^c^	0.29	0.29^d^	0.43	0.14
8	2.24	1.85 ± 0.25^a^	2.32		2.34^c^	0.25	0.25^d^	0.38	0.38
9	2.42	1.95 ± 0.25^a^	2.49		2.49^c^	0.22	0.44^d^	0.56	0.11
10	2.36	2.00 ± 0.25^a^	2.63		2.69^c^	0.40	0.60^d^	0.30	0.50
11	2.54	1.95 ± 0.25^a^	2.70		2.78^c^	0.36	0.60^d^	0.45	0.45
12	2.63	2.00 ± 0.30^a^	2.89		2.83^c^	0.50	0.50^d^	0.42	0.42
13	2.62	2.25 ± 0.30^a^	2.70	2.80^b^	2.94^c^	0.46	0.60^d^	0.23	0.38
14	2.71		2.93		2.99^c^	0.43	0.60^d^	0.50	0.36
15	3.12		3.05		3.14^c^	0.27	0.50^d^	0.47	0.47
16	2.35		2.40			0.50	0.40^d^	0.44	0.56
17	3.16		3.04			0.24	0.40^d^	0.53	0.41
18	3.12		3.18			0.44	0.30^d^	0.50	0.39
19	3.19		3.27			0.42	0.30^d^	0.37	0.37
20	3.25		3.21			0.30	0.40^d^	0.45	0.35

^a^ref. 27.

^b^ref. 24.

^c^ref. 26.

^d^ref. 13.
