# Supplementary material for: Insights into the geometries, electronic and magnetic properties of neutral and charged palladium clusters
Source: Sci Rep. 2016 Jan 22;6:19656. doi: 10.1038/srep19656 (PMC4726383; doi:10.1038/srep19656)
Supplement: Supplementary Information [file srep19656-s1.pdf]

Insights into the geometries, electronic and magnetic properties of neutral and charged palladium clusters

Xiaodong Xing[1,2], Andreas Hermann[3], Xiaoyu Kuang[1,*], Meng Ju[1], Cheng Lu[2,4,*], Yuanyuan Jin[1], Xinxin Xia[1], and George Maroulis[5,*]

[1]Institute of Atomic and Molecular Physics, Sichuan University, Chengdu 610065, China

[2]Department of Physics, Nanyang Normal University, Nanyang 473061, China

[3]Centre for Science at Extreme Conditions and SUPA, School of Physics and Astronomy, The University of Edinburgh, Edinburgh EH9 3JZ, United Kingdom

[4]Beijing Computational Science Research Center, Beijing 100084, China

[5]Department of Chemistry, University of Patras, GR-26500 Patras, Greece

*Correspondence author. E-mail: scu_kuang@163.com (Xiao-Yu Kuang), lucheng@calypso.cn (Cheng Lu) and maroulis@upatras.gr (George Maroulis)

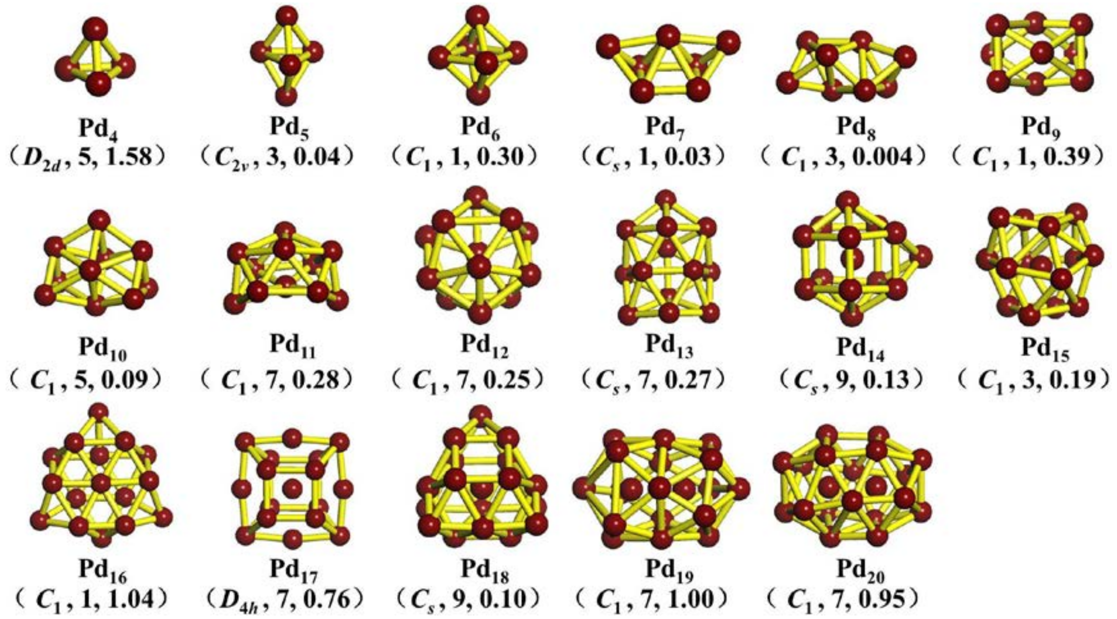

Fig S1. The metastable isomers of Pd_n (n = 4-20) clusters, together with spin multiplicity, point symmetry and relative energies.

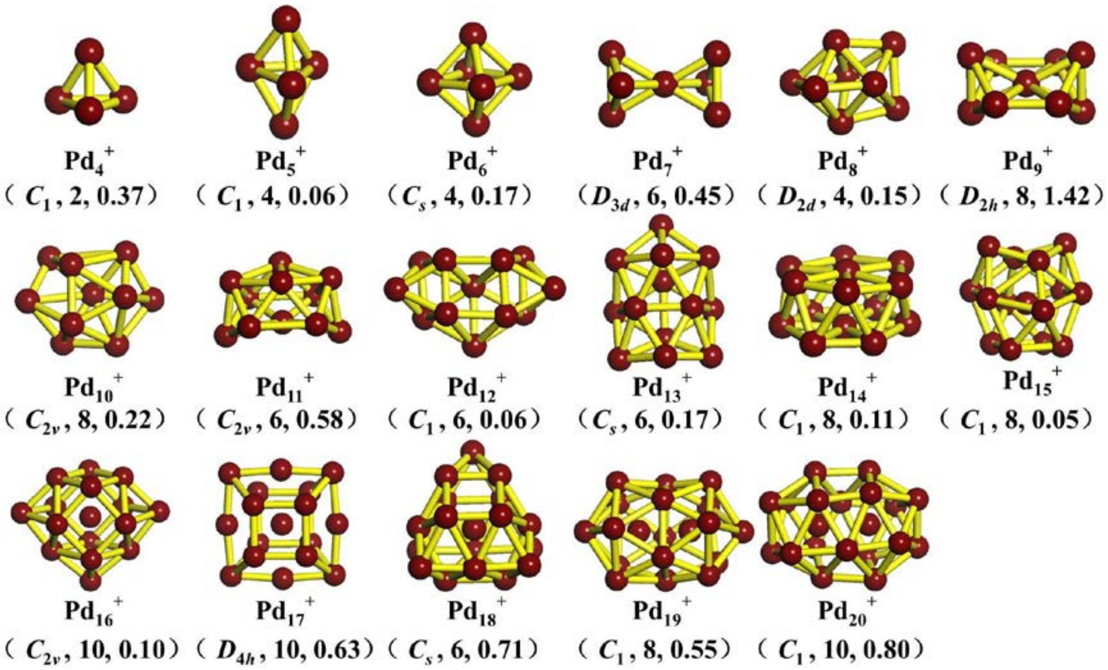

Fig S2. The metastable isomers of Pd_n^+ (n = 4-20) clusters, together with spin multiplicity, point symmetry and relative energies.

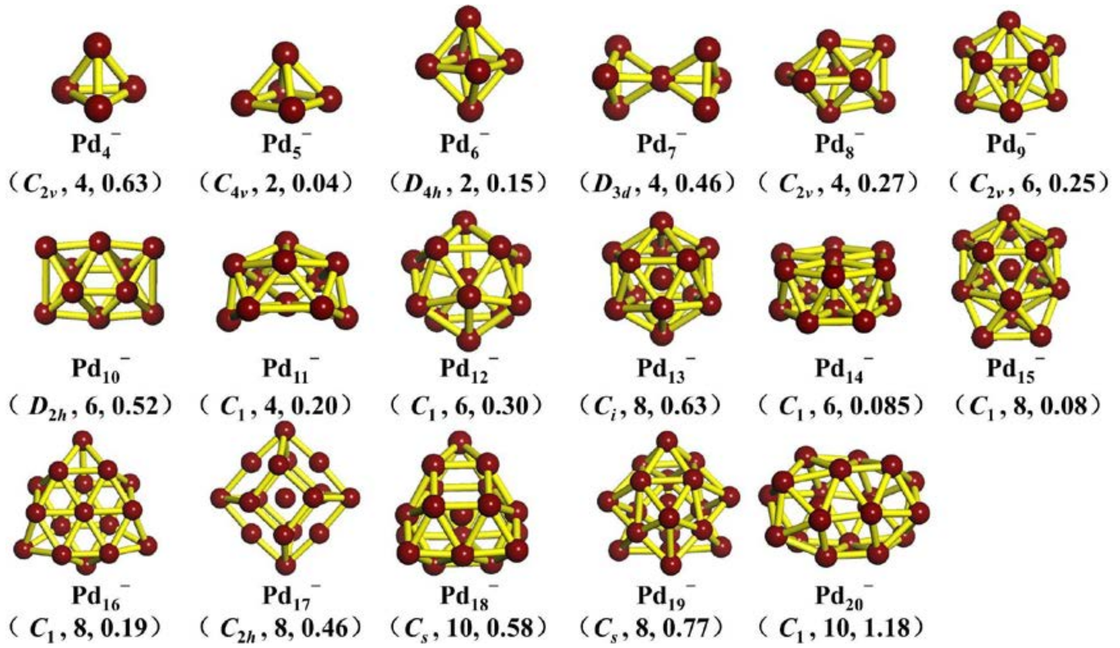

Fig S3. The metastable isomers of Pd_n^- (n = 4-20) clusters, together with spin multiplicity, point symmetry and relative energies.

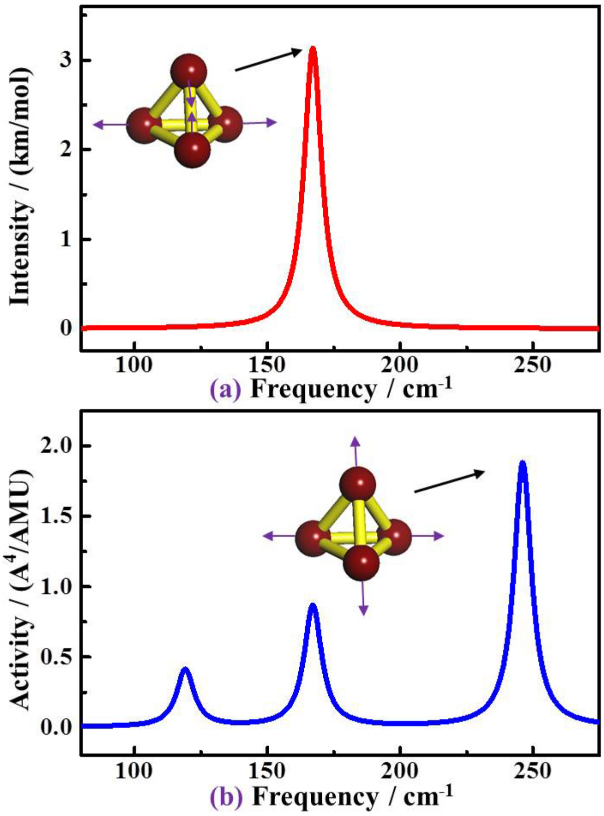

Fig S4. The infrared (a) and Raman (b) spectra of the Pd_4^+ cluster. Insets show the frequency modes corresponding to the highest activity or intensity.

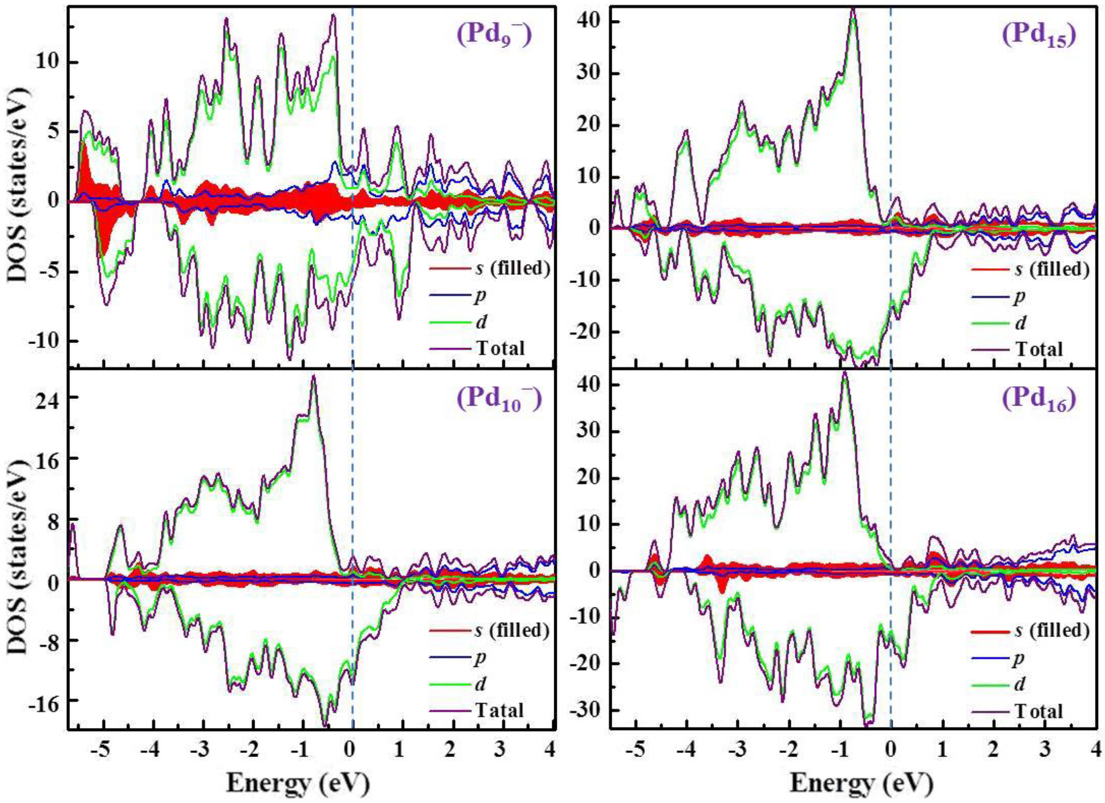

Fig S5. Calculated DOS of Pd_9^-, Pd_{10}^-, Pd_{15} and Pd_{16} clusters. The Fermi level is shifted to zero.

	State	Symm.	E_b	E_{gap}		State	Symm.	E_b	E_{gap}		State	Symm.	E_b	E_{gap}
Pd_2	$^3S_{gg}$	$D_{\infty h}$	0.48	1.33	Pd_2^+	$^2S_{gu}$	$D_{\infty h}$	1.06	2.70	Pd_2^-	$^2S_{gu}$	$D_{\infty h}$	1.17	0.93
Pd_3	$^3A''$	C_s	0.84	1.54	Pd_3^+	$^2B_2$	C_{2v}	1.20	1.24	Pd_3^-	$^2B_2$	C_{2v}	1.50	2.12
Pd_4	$^3A''$	C_s	1.25	1.75	Pd_4^+	$^4A_1$	T_d	1.66	2.58	Pd_4^-	$^2A_2$	D_{2d}	1.59	1.47
Pd_5	$^3B_2$	C_{2v}	1.32	1.85	Pd_5^+	$^4A'$	C_s	1.70	1.73	Pd_5^-	$^2B_1$	C_{2v}	1.65	1.39
Pd_6	$^3B_2$	C_{2v}	1.41	1.46	Pd_6^+	^4B_u	C_{2h}	1.75	0.95	Pd_6^-	$^2B_{3g}$	D_{2h}	1.71	1.63
Pd_7	^3A	C_1	1.44	1.60	Pd_7^+	$^4A_2'$	D_{5h}	1.68	1.14	Pd_7^-	$^2A_2'$	D_{5h}	1.69	1.36
Pd_8	^3A	C_1	1.46	1.38	Pd_8^+	^4A	C_1	1.76	1.61	Pd_8^-	^4A	C_1	1.74	1.39
Pd_9	^3A	C_1	1.51	0.74	Pd_9^+	^6A	C_1	1.78	1.47	Pd_9^-	^2A	C_1	1.78	1.52
Pd_{10}	^5A	C_1	1.55	1.40	Pd_{10}^+	$^4B_1$	C_{2v}	1.76	1.00	Pd_{10}^-	^6A	C_1	1.78	1.25
Pd_{11}	^5A	C_2	1.59	1.34	Pd_{11}^+	^6A	C_1	1.80	1.34	Pd_{11}^-	^6A	C_1	1.82	1.34
Pd_{12}	^7A	C_1	1.61	1.28	Pd_{12}^+	^6A	C_1	1.80	1.25	Pd_{12}^-	^6A	C_1	1.83	0.83
Pd_{13}	^7A	C_1	1.65	1.31	Pd_{13}^+	^4A	C_1	1.82	0.75	Pd_{13}^-	^6A	C_1	1.85	1.25
Pd_{14}	^7A	C_1	1.66	0.59	Pd_{14}^+	$^8A''$	C_s	1.84	0.96	Pd_{14}^-	^6A	C_1	1.86	0.92
Pd_{15}	^5A	C_1	1.67	1.13	Pd_{15}^+	^8A	C_1	1.84	1.02	Pd_{15}^-	^8A	C_1	1.88	0.79
Pd_{16}	$^9A''$	C_s	1.71	1.12	Pd_{16}^+	$^8A'$	C_s	1.85	0.93	Pd_{16}^-	$^{10}A''$	C_s	1.86	0.31
Pd_{17}	^5A	C_1	1.73	1.23	Pd_{17}^+	^{10}A	C_1	1.88	1.26	Pd_{17}^-	^8A	C_1	1.92	1.18
Pd_{18}	^9A	C_1	1.77	1.16	Pd_{18}^+	^{10}A	C_1	1.90	1.13	Pd_{18}^-	^8A	C_1	1.95	1.22
Pd_{19}	^9A	C_1	1.80	1.16	Pd_{19}^+	^8A	C_1	1.91	1.17	Pd_{19}^-	^8A	C_1	1.97	1.16
Pd_{20}	^7A	C_1	1.80	0.85	Pd_{20}^+	^{10}A	C_1	1.93	1.20	Pd_{20}^-	^8A	C_1	1.97	0.88

Table S1. The electronic states, symmetries, averaged binding energy (E_b, eV), and HOMO–LUMO energy gap (E_{gap}, eV) for the lowest-energy structures of $Pd_n^{0/+/-}$ (n = 2-20) clusters.

Final cluster	Initial clusters																		
	2	3	4	5	6	7	8	9	10	11	12	13	14	15	16	17	18	19	20
1	0.96	1.55	2.49	1.61	1.84	1.61	1.63	1.92	1.84	1.99	1.90	2.15	1.78	1.86	2.21	2.13	2.46	2.24	1.89
2		1.55	3.08	3.15	2.51	2.50	2.28	2.59	2.80	2.88	2.93	3.09	2.97	2.68	3.11	3.37	3.62	3.73	3.17
3			2.49	3.15	3.45	2.57	2.58	2.66	2.89	3.25	3.23	3.54	3.32	3.29	3.34	3.69	4.28	4.31	4.08
4				1.61	2.51	2.57	1.72	2.02	2.01	2.39	2.66	2.89	2.83	2.70	3.00	2.98	3.66	4.03	3.72
5					1.85	2.50	2.58	2.02	2.24	2.38	2.67	3.19	3.05	3.07	3.28	3.51	3.82	4.27	4.30
6						1.61	2.28	2.66	2.01	2.38	2.44	2.98	3.12	3.07	3.43	3.56	4.12	4.20	4.32
7							1.63	2.59	2.89	2.39	2.67	2.98	3.15	3.38	3.66	3.94	4.41	4.74	4.49
8								1.92	2.80	3.20	2.66	3.19	3.12	3.38	3.95	4.15	4.77	5.01	5.01
9									1.84	2.88	3.23	2.89	3.05	3.07	3.66	4.15	4.69	5.08	4.99
10										1.99	2.93	3.54	2.83	3.07	3.43	3.94	4.77	5.08	5.14

Table S2. Fragmentation energies (eV) of Pd_n (n = 2-20) clusters.

Final	Initial clusters																		
cluster	2	3	4	5	6	7	8	9	10	11	12	13	14	15	16	17	18	19	20
1	2.35	2.16	1.85	1.84	1.99	1.59	2.05	2.08	1.77	2.15	1.97	2.14	1.87	2.27	1.44	2.93	2.42	2.31	1.95
2		3.55	3.07	2.76	2.91	2.66	2.72	3.21	2.92	2.99	3.20	3.17	3.06	3.18	2.75	3.41	4.39	3.77	3.30
3			3.87	3.38	3.23	2.97	3.19	3.27	3.45	3.54	3.44	3.79	3.50	3.78	3.08	4.13	4.28	5.15	4.17
4				3.24	2.90	2.35	2.56	2.80	2.57	3.13	3.05	3.09	3.18	3.28	2.73	3.52	4.06	4.10	4.61
5					3.63	2.89	2.80	3.03	2.96	3.12	3.50	3.57	3.35	3.83	3.10	4.04	4.32	4.75	4.43
6						3.39	3.12	3.05	2.97	3.28	3.26	3.79	3.59	3.77	3.42	4.18	4.61	4.78	4.85
7							3.86	3.60	3.23	3.53	3.66	3.79	4.06	4.25	3.60	4.74	4.99	5.31	5.12
8								4.32	3.75	3.76	3.89	4.17	4.03	4.69	4.06	4.89	5.53	5.67	5.63
9									4.18	4.00	3.83	4.11	4.13	4.38	4.21	5.07	5.39	5.92	5.70
10										4.51	4.15	4.13	4.14	4.55	3.98	5.30	5.64	5.86	6.02
11											4.51	4.30	4.01	4.42	4.00	4.92	5.72	5.96	5.82
12												4.75	4.27	4.38	3.96	5.03	5.43	6.13	6.01
13													4.47	4.39	3.67	4.74	5.30	5.59	5.93
14														4.96	4.05	4.82	5.38	5.83	5.76
15															4.54	5.12	5.38	5.82	5.91
16																5.26	5.33	5.48	5.57
17																	5.56	5.52	5.31
18																		5.41	5.01
19																			5.12

Table S3. Fragmentation energies (eV) of Pd_n^- (n = 2-20) clusters.

Final cluster	Initial clusters																		
	2	3	4	5	6	7	8	9	10	11	12	13	14	15	16	17	18	19	20
1	2.11	1.50	3.02	1.87	1.98	1.26	2.36	1.89	1.59	2.27	1.80	1.97	2.10	1.84	1.97	2.46	2.17	2.07	2.27
2		2.65	3.56	3.93	2.89	2.28	2.67	3.29	2.52	2.90	3.11	2.81	3.11	2.98	2.85	3.47	3.67	3.28	3.38
3			4.13	3.15	4.36	2.60	3.09	3.00	3.34	3.24	3.16	3.53	3.36	3.40	3.41	3.76	4.09	4.19	4.00
4				3.51	3.37	3.13	2.48	2.49	2.11	3.12	2.55	2.63	3.14	2.72	2.88	3.38	3.44	3.67	3.97
5					3.86	3.01	3.88	2.74	2.47	2.76	3.30	2.90	3.12	3.37	3.07	3.72	3.93	3.90	4.32
6						3.28	3.53	3.92	2.49	2.89	2.71	3.42	3.16	3.11	3.49	3.68	4.05	4.15	4.32
7							4.03	3.81	3.90	3.15	3.08	3.07	3.91	3.39	3.47	4.34	4.24	4.51	4.81
8								4.28	3.77	4.53	3.31	3.41	3.54	4.12	3.72	4.30	4.87	4.67	5.14
9									3.96	4.11	4.42	3.36	3.59	3.46	4.17	4.26	4.54	5.02	5.02
10										4.38	4.07	4.54	3.62	3.59	3.58	4.78	4.59	4.77	5.45
11											4.19	4.05	4.65	3.47	3.57	4.05	4.96	4.67	5.05
12												4.26	4.25	4.59	3.54	4.13	4.32	5.13	5.03
13													4.21	3.94	4.41	3.85	4.15	4.24	5.25
14														4.27	4.13	5.09	4.24	4.44	4.73
15															4.38	4.73	5.40	4.44	4.84
16																4.63	4.69	5.26	4.51
17																	4.68	4.64	5.40
18																		4.29	4.45
19																			4.32

Table S4. Fragmentation energies (eV) of Pd_n^+ (n = 2-20) clusters.

Size	Neutral	Cation	Anion
2	0.000	0.000	0.000
3	0.103	0.502	0.001
4	0.039	0.000	0.000
5	0.001	0.130	0.078
6	0.002	0.000	0.000
7	0.173	0.000	0.000
8	0.000	0.249	0.417
9	0.085	0.077	0.124
10	0.278	0.031	0.405
11	0.054	0.638	0.179
12	0.233	0.276	0.122
13	0.206	0.734	0.371
14	0.003	0.358	0.464
15	0.219	0.281	0.340
16	0.478	0.069	0.156
17	0.484	0.576	0.360
18	0.695	1.099	0.382
19	0.405	0.665	0.221
20	1.080	1.186	0.963

Table S5. The electric dipole moments (μ) of $Pd_n^{0/+/–}$ (n = 2-20) clusters.

Cluster	Neutral		Cation	Anion
	This work	Theo.[a]		
2	42.11	29.96	18.30	89.44
3	38.85	35.62	25.34	62.64
4	32.70	34.03	25.39	52.75
5	32.41	33.13	26.06	46.47
6	31.43	31.25	24.74	41.86
7	30.49	32.08	26.93	42.39
8	30.53	31.36	26.25	47.30
9	34.05	31.51	29.67	42.56
10	34.28	31.54	28.40	43.85
11	34.12	32.25	30.32	42.55
12	34.52	31.85	29.96	41.12
13	34.53	30.81	30.61	40.61
14	34.35	33.30	31.56	39.93
15	33.62	35.10	30.26	39.08
16	33.86	34.16	30.40	39.86
17	33.41	34.03	30.39	38.06
18	33.21	34.25	30.37	37.58
19	32.94	34.37	30.49	36.83
20	32.87	34.16	30.44	36.56

Table S6. The mean polarizability per atom ($e^2a_0^2E_h^{-1}$/atom) of $Pd_n^{0/+/-}$ (n = 2-20) clusters, together with the available theoretical data for comparison. [a]Ref. 1.

References

1. Ma, L., Wang, J. & Wang, G. Dipole polarizabilities of Pd_n (n = 2-25) clusters. *Eur. Phys. J. D* **67,** 6-14 (2013).
